# Prediction of Rotator Cuff Muscle Fibre Orientations Using a Population‐Averaged Atlas Generated With Anatomical and Diffusion‐Weighted Magnetic Resonance Images

**DOI:** 10.1002/nbm.70119

**Published:** 2025-08-31

**Authors:** Yilan Zhang, Robert Lloyd, Robert D. Herbert, Lynne E. Bilston, Bart Bolsterlee

**Affiliations:** ^1^ Neuroscience Research Australia (NeuRA) Sydney Australia; ^2^ Graduate School of Biomedical Engineering University of New South Wales Sydney Australia; ^3^ School of Biomedical Sciences University of New South Wales Sydney Australia; ^4^ School of Mechanical, Medical and Process Engineering Queensland University of Technology Brisbane Australia

**Keywords:** diffusion‐weighted imaging, fibre orientation, multichannel registration, musculoskeletal imaging, rotator cuff muscles

## Abstract

Measurements of muscle architecture are crucial for understanding muscle function but are often difficult to obtain in human muscles in vivo. This study aimed to create population‐averaged atlases of human rotator cuff muscle shape and muscle fibre orientations from anatomical magnetic resonance images (MRI) and diffusion‐weighted images (DWI) and to utilise these atlases to predict muscle fibre orientations from anatomical MRI data alone. An image registration framework was applied to coregister anatomical MRI and DWI data of 11 male and 9 female subjects into sex‐specific common spaces, forming the basis for the atlases. The accuracy of registration was quantified using Dice coefficients, angular correlation coefficients (ACCs) and angular differences. The same metrics were used to assess the capability of the atlases to predict fibre orientations for subjects not included in the atlas construction, via leave‐one‐out cross‐validation. The results showed that individual male and female image data were accurately registered into their respective atlas spaces, with high Dice coefficients (0.888 ± 0.002 for males, 0.856 ± 0.021 for females) and consistent angular alignment as evidenced by the ACCs and angular differences. Predicted fibre orientations for out‐of‐sample subjects closely matched those derived from DWI images, exhibiting improved smoothness and coverage (ACC: 0.909 ± 0.011 for males, 0.942 ± 0.011 for females; angular difference: 13.8° ± 1.3° for males, 11.2° ± 1.2° for females). These findings demonstrate that population‐averaged atlases enhance muscle architecture reconstructions and enable the accurate prediction of muscle fibre orientations using only anatomical MRI scans in younger individuals without shoulder injuries.

## Graphical abstract

In this study, we developed population‐averaged atlases of human rotator cuff muscles from anatomical and diffusion‐weighted MRI data. The atlases accurately predict muscle fibre orientations from anatomical MRI scans, enabling enhanced musculoskeletal modelling and improved representation of muscle architecture.

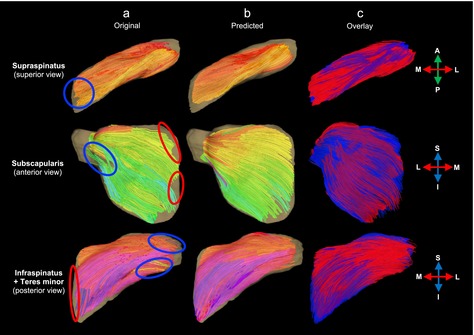

**Abbreviations**
ACC: angular correlation coefficient
DWI: diffusion‐weighted imaging
FOD: fibre orientation distributions
LOOCV: leave‐one‐out cross‐validation
SH: spherical harmonics
SSM: statistical shape modelling
SyN: symmetric normalisation

## 1 Introduction

Skeletal muscle architecture, the macrostructural arrangement of muscle fibres in the muscle belly, is the primary determinant of a muscle's capacity to generate force and change length [1]. Though broadly consistent across individuals, muscle architecture can adapt to exercise [2, 3], ageing [4, 5] and disease [6, 7, 8]. Measurements of muscle‐ and subject‐specific architecture are therefore useful for understanding the functional consequences of muscle adaptations.

The human rotator cuff, comprising the supraspinatus, subscapularis, infraspinatus and teres minor muscles, is crucial for upper limb mobility and glenohumeral joint stability. Rotator cuff injuries are common—they constitute up to half of all significant shoulder injuries seen in some clinical contexts [9, 10]—and can be difficult to treat. Computational models can be used to predict the biomechanical consequences of rotator cuff tears on muscle and joint function [11, 12] or guide the design of implants in shoulder arthroplasty procedures [13]. However, interindividual anatomical variations in muscle architecture are usually not reflected in these models because of the challenge of acquiring subject‐specific measurements of rotator cuff muscle architecture in vivo [14]. Computational models and surgical planning tools might be more useful if they could incorporate subject‐specific measurements of rotator cuff muscle architecture.

Diffusion‐weighted imaging (DWI) enables measurement of human rotator cuff muscle architecture in three dimensions by tracking fibre tracts along principal eigenvectors of diffusion tensors throughout a muscle [15, 16]. However, DWI‐based reconstructions are sensitive to noise and image artifacts inherent in DWI data, which can distort the path of fibre tracts and create regions in the muscle that contain few fibres. Moreover, the long DWI acquisitions (~10 min) are not part of standard clinical shoulder imaging protocols. New methods are needed to facilitate the widespread use of muscle architecture reconstructions from MRI data.

Population‐based approaches like statistical shape modelling (SSM) offer a potential solution, because they can be used to map a template (e.g., the mean shape across a population obtained through coregistration of images) to an individual subject to the desired level of detail required for the application and allowed for by the available data [17, 18]. The relatively few attempts to apply population‐based approaches to skeletal muscles (e.g., facial muscles [19], levator ani [20], soleus [21] and hamstrings [22]) only modelled the shape (outer surface) of the muscle but did not model muscle fibre orientations. One recent study included muscle fibre orientations in a population‐averaged framework [23] but only modelled a single muscle per model and did not use information about muscle fibre orientations for registration, potentially limiting the accuracy of the population‐averaged model.

Population‐averaged atlases built using multichannel registration, which are already popular in neuroimaging [24, 25, 26, 27], introduce novel possibilities for muscle architecture analysis. First, these atlases may enhance the representation of individual muscle architecture by aggregating information across the population, potentially reducing noise and image artifacts typically present in individual scans and enhancing the robustness and reliability of muscle architecture reconstructions. Second, given the limited interindividual variability in rotator cuff muscle architecture reported in previous studies [16], these atlases could potentially be used to predict individual muscle architecture from muscle shape alone, simplifying the generation of high‐quality muscle architecture reconstructions.

The goals of this study were, therefore, to (1) develop population‐averaged atlases of human rotator cuff muscles that combine muscle shape data derived from anatomical MRI with fibre orientation data derived from DWI and (2) demonstrate the use of the atlases to (2a) predict muscle fibre orientations from anatomical MRI scans alone and (2b) enhance the smoothness of fibre tracts and fill in areas of sparse data that often occur in subject‐specific scans due to noise and image artifacts inherent in DWI data.

## 2 Methods

### 2.1 Participants

Study procedures were approved by the UNSW Human Research Ethics Committee (HREC approval HC200971). Each participant was informed about the study procedures and provided their written consent before participation. The study involved magnetic resonance imaging (MRI) of the right shoulder of 11 male participants (age: 27 ± 6 years, height: 176 ± 7 cm, weight: 71 ± 9 kg, values are mean ± standard deviation) and 9 female participants (age: 29 ± 7 years, height: 165 ± 4 cm, weight: 53 ± 5 kg). People with symptoms or a recent history of shoulder pathology were excluded from participating.

Some of the data used in this study have been used in previous studies on rotator cuff muscle architecture [15, 16].

### 2.2 MRI Acquisitions and Processing

A brief overview of image acquisition and processing will be provided here. A more detailed description can be found elsewhere [15, 16].

All MR images were acquired at 3T (Philips Ingenia CX, Philips Healthcare, Best, The Netherlands) with a dS torso/anterior coil and dS posterior coil (28 channels in total). The protocol consisted of an mDixon scan and two diffusion‐weighted scans covering the proximal and distal rotator cuff musculature, respectively. We chose to acquire DWI scans in two stacks, as acquiring a single DWI scan with sufficient spatial resolution and coverage would require 25–30 min of scan time, which would increase the risk of subject motion and image artefacts. Using two shorter, targeted acquisitions provided a practical balance between spatial coverage, scan duration and image quality. The scan parameters for the mDixon images were as follows: a two‐point 3D T1‐weighted fast field echo (FFE) sequence with TR/TE1/TE2 = 6.0/3.5/4.6 ms, field of view (FOV) = 240 mm, voxel size = 1 × 1 × 2 mm, acquisition matrix = 240 × 198 (reconstructed to 320 × 264), 210 slices, number of signal averages = 2 and total scan time = 6 min. The DWI protocol was based on a single‐shot echo‐planar imaging (EPI) sequence with TR/TE = 3000/46 ms, FOV = 190 mm, voxel size = 2.5 × 2.5 × 5 mm, slice gap = 0.5 mm, acquisition matrix = 76 × 76 (reconstructed to 240 × 240), 24 slices and 12 diffusion encoding directions distributed on a hemisphere. Images were acquired at *b* = 0 and *b* = 500 s/mm^2^, with 9 signal averages and a total scan time of approximately 13 min for both scans combined. To correct for susceptibility‐induced geometric and intensity distortions [28, 29], two *b* = 0 images with reversed phase‐encoding directions were acquired and subsequently processed using standard distortion correction methods.

Segmentation of rotator cuff muscles and bones (humerus, scapula and clavicle) was initially performed manually on a subset of 12 mDixon scans from six males and six females, obtained previously in our laboratory using the same image protocol, after which a deep learning model (nnU‐net [30]) was trained for automatic segmentation of the remaining scans. The predicted segmentations were visually verified and adjusted when needed. In four out of 20 scans, the boundary between the infraspinatus and teres minor muscles was not clearly visible. These muscles were therefore grouped together and collectively analysed for all participants. Previous analysis demonstrated excellent intrarater reliability of segmentation (average intraclass correlation coefficient across muscles of 0.97 [16]).

DWI scans were denoised using a Marchenko–Pastur principal component analysis filter [31, 32] corrected for eddy current‐ and possible motion‐induced distortions using functions TOPUP and EDDY from FSL [33], implemented in MRtrix [34]. The processed DWI scans were then upsampled using linear interpolation to match the spatial dimensions of the mDixon scan and combined into a single DWI image set covering the entire rotator cuff. Rigid registration via the FLIRT tool from FSL [35, 36] was performed to correct for small misalignments between the mDixon and DWI images within subjects. Registration accuracy was visually confirmed in ITK‐SNAP [37].

To increase anatomical contrast and improve subsequent image registration, the mDixon scans, stitched DWI scans and segmented masks were linearly upsampled to an isotropic voxel size of 0.75 × 0.75 × 0.75 mm.

### 2.3 Construction of Population‐Averaged Atlases

The construction of population‐averaged atlases was a multistep process involving estimation of fibre orientation distributions (FODs) from DWI data, concurrent multichannel registration of individual mDixon images and the FODs into a common reference frame and averaging across subjects to construct a cohesive atlas. In the initial phase of this study, we tried to construct a single multichannel population‐averaged atlas for both males and females. However, it proved difficult to achieve accurate registrations when male and female data were combined into a single atlas, even when using affine and non‐rigid transformations, presumably because large differences in muscle volumes between males and females led to difficulties in achieving accurate alignment. Therefore, we created sex‐specific atlases for males (*n* = 11) and females (*n* = 9).

#### 2.3.1 FOD Estimation From DWI

FODs were derived from DWI data using algorithms included in MRtrix [38, 39]. Although FODs can describe multiple fibre orientations within a voxel, previous investigations have shown that muscle fibre orientations within a voxel can be represented well by a single orientation [16]. We therefore limited the maximum spherical harmonics (SH) degree to two in the FOD estimation process, retaining only the principal diffusion direction within a voxel, analogous to a single‐fibre diffusion tensor model. The response function [38], characterising the diffusion signal intensity based on the orientation of a single fibre bundle under magnetic gradients, served as a kernel for spherical deconvolution. The FOD estimation was then performed on DWI images with constrained spherical deconvolution using the response function as an input [39]. Segmented muscle masks were used to restrict FOD estimation to the rotator cuff muscles.

#### 2.3.2 Multichannel Registration

Data from *n* − 1 subjects (*n* = 11 for males, *n* = 9 for females) were used for the creation of population‐averaged atlases. A leave‐one‐out cross‐validation method was applied, where 11 male atlases and 9 female atlases were created, each based on *n* − 1 subjects, where the left‐out (out‐of‐sample) subject was used for evaluation purposes (see Section 2.4).

**FIGURE 1:**
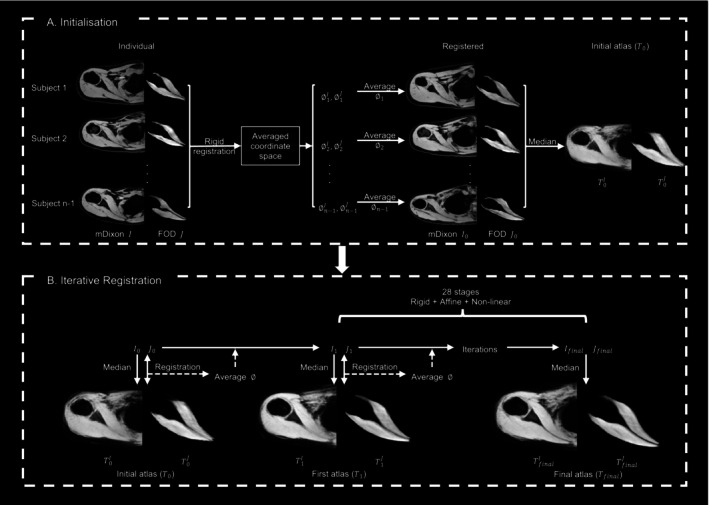
Schematic overview of multichannel registration pipeline. (A) The process was initialised by aligning individual mDixon *I* and FOD images *J* to a common space to create an unbiased initial atlas $T_{0}$, obtained by taking the median of registered images ($I_{0}$ and $J_{0}$). (B) Each subject's images underwent a series of rigid, affine and non‐linear registrations, iteratively for 28 stages. At each stage, the median of registered mDixon $I_{k}$ (k∈ [1, 28]) and FOD $J_{k}$ images were computed to update the atlas $T_{k}$.

The registration pipeline is based on an intensity‐based multichannel registration framework implemented in MRtrix. The input channels for each subject consist of all four mDixon images (water only, fat only, in‐phase and out‐of‐phase) and six FOD image volumes, containing the parameters of the two‐degree‐of‐freedom SH describing the fibre orientations [39]. We used all four mDixon images to use the complementary information provided by each image type in the registration. Although in‐phase images offer good general structural detail, water‐only and fat‐only images help delineate muscle boundaries and surrounding fat, and out‐of‐phase images assist in identifying subtle tissue interfaces. Masks of rotator cuff muscles and the scapula were used as inputs to target the registration to the primary region of interest. The pipeline consists of two steps: initialisation and iterative registration (see Figure 1). All registrations used symmetric normalisation (SyN) Demons [40] with the sum of squared difference (SSD) metric and reorientation of FOD using apodised point spread functions [41].

##### 2.3.2.1 Initialisation

The initialisation step created an unbiased initial atlas to serve as a reference for the subsequent registration process. First, the average coordinate space of all input FODs was calculated. Then, for each subject, the mDixon *I* and FOD *J* channels were rigidly registered to this space, respectively. Each channel's transformations were then averaged and applied to the mDixon and FOD data. The preliminary set of mDixon $T_{0}^{I}$ and FOD $T_{0}^{J}$ atlases was then generated by computing the median image intensity across all registered mDixon $I_{0}$ and FOD $J_{0}$ volumes, respectively. We empirically found that using the median instead of the mean of image intensities gave clearer structure boundaries.

##### 2.3.2.2 Iterative Registration

Unbiased population‐averaged atlases were constructed through iterative registration. In each iteration, the median intensity values of the registered mDixon and FOD volumes were computed separately to continually update and refine their respective atlases. This process involved iterating over 28 stages, organised into a sequence of 6 rigid, 6 affine and 16 non‐linear registration stages. The atlas generation at each stage *k* can be summarised in the following steps:

1. For each subject, mDixon $I_{k-1}$ (k∈ [1, 28]) and FOD $J_{k-1}$ channels were registered to their corresponding updated atlases $T_{k-1}^{I}$ and $T_{k-1}^{J}$ (to preliminary atlases $T_{0}$ in the first stage) using rigid, affine or non‐linear registration based on the stage.
2. Resulting transformations (rigid and affine registrations) or displacement fields (non‐linear registration) from each channel were averaged (∅) and applied to mDixon and FOD data.
3. The median of registered mDixon $I_{k}$ and FOD $J_{k}$ volumes were computed to update their atlases $T_{k}^{I}$ and $T_{k}^{J}$.

A multiresolution pyramid with default scale values in MRtrix (Table 1) was used to perform registration at different resolution levels, starting with down‐sampled images (scale < 1) and progressively working towards original high‐resolution images (scale = 1). The use of this multiresolution pyramid enhances efficient image registration by focusing on large structures first, followed by focusing on finer details [42].

**TABLE 1:** Summary of registration parameters.

Stage no.	Type of registration	Number of iterations^a^	SH degrees	Multiresolution pyramid^b^
1–6	Rigid	1000	2	0.3, 0.4, 0.6, 0.8, 1.0, 1.0
7–12	Affine	1000	2	0.3, 0.4, 0.6, 0.8, 1.0, 1.0
13–28	Nonlinear	500	2	0.3, 0.4, 0.5, 0.6, 0.7, 0.8, 0.9, 1.0, 1.0, 1.0, 1.0, 1.0, 1.0, 1.0, 1.0, 1.0

Abbreviation: SH = spherical harmonic.

^a^ The number of registration iterations used within each stage before updating the atlas.

^b^ The list of scale values that represents the degree to which the original image was downsampled in each iteration.

The displacement field obtained at each non‐linear iteration was smoothed using a Gaussian filter with a standard deviation of twice the voxel size of the iteration's resolution.

After registration, the output consisted of three components:

1. A population‐averaged mDixon atlas $T_{\text{final}}^{I}$ and a corresponding FOD atlas $T_{\text{final}}^{J}$ in a common coordinate space (atlas space);
2. Individual subject image data, specifically, mDixon images $I_{r}$ ($r\in \left[1,n-1\right]$), FODs $J_{r}$ and masks of muscles and the scapula, each registered and aligned to the atlas space; and
3. For each subject, a displacement field $\emptyset_{r}$ that warps subject images to the atlas space. This included both linear (rigid and affine) and non‐linear displacements.

We computed the atlas mask by calculating the median of all registered subject masks in atlas space.

#### 2.3.3 Evaluation

##### 2.3.3.1 Qualitative Evaluation

Qualitative evaluation included reconstruction, visual inspection and comparison of fibre tractography of all muscles to visually assess fibre orientations. Fibre tracts were reconstructed from each subject's FODs and from the population‐averaged FOD atlases, using a deterministic tractography algorithm based on spherical deconvolution [43]. The tracts were dynamically seeded using the SIFT model within MRtrix [44], a mechanism designed to improve the evenness of tract density distribution. In each muscle, a total of 3000 fibre tracts were generated using fibre track settings previously used for rotator cuff muscle architecture reconstructions [15, 16]: integration step size = 1.0 mm; 0.1 ≤ fractional anisotropy ≤ 0.5; maximum turning angle between successive steps > 15°; 25 mm ≤ tract length ≤ 200 mm.

##### 2.3.3.2 Quantitative Evaluation

The multichannel registration process was validated using leave‐one‐out cross‐validation (LOOCV), independently for male and female atlases. Thus, 11 male and 9 female atlases were created, each based on a different subset of 10 and 8 subjects, respectively.

All atlases were visually inspected to ensure proper alignment between channels and to verify the anatomical fidelity of structures. We then quantitatively evaluated the performance of multichannel registration using three metrics per muscle. The spatial alignment accuracy was measured by the Dice coefficient [45] between the atlas mask and the subject‐specific mask warped to the atlas. A Dice coefficient closer to 1 indicates a greater degree of alignment between the spatial locations of the masks. The angular alignment between estimated and subject‐specific fibre orientations was quantified by calculating the angular correlation coefficient (ACC) [46] between the FOD atlas and the FOD of each subject warped to the atlas space. The ACC was determined for each muscle on a voxel‐by‐voxel basis as

(1) $$\mathrm{ACC}=\frac{\sum_{m=-l}^{l}a_{l}^{m}b_{l}^{m}}{{\left(\sum_{m=-l}^{l}{\left(a_{l}^{m}\right)}^{2}\;\right)}^{\frac{1}{2}}{\left(\sum_{m=-l}^{l}{\left(b_{l}^{m}\right)}^{2}\;\right)}^{\frac{1}{2}}}$$

where $a_{l}^{m}$ and $b_{l}^{m}$ represent the coefficient for the SH function of degree l (l=2 in this study) and order m of the FOD atlas and the warped FOD, respectively.

To quantify the alignment between population‐averaged and subject‐specific fibre orientations, we first calculated the principal diffusion direction within each voxel of the FODs using the sh2peaks function in MRtrix. Subsequently, we calculated the 3D angular difference between each of the peak direction vectors for the atlas and the corresponding vectors warped from the subject's FOD. Although ACC provides a dimensionless measure of similarity in orientation distribution, angular difference provides a measure of muscle fibre orientation alignment accuracy expressed in a familiar metric (degrees). The median ACCs and median angular difference were used to summarise the alignment between population‐averaged and subject‐specific fibre orientations for a muscle. Histograms illustrating the distributions can be found in the Supporting Information.

The mean Dice coefficient, ACC and angular difference, along with their respective standard deviations, were calculated for each individual case from the LOOCV.

### 2.4 Prediction of Fibre Orientations From Anatomical MRI

To demonstrate the use of the atlases to predict muscle fibre orientations from anatomical mDixon scans alone, we compared fibre orientations obtained from a subject's DWI data (ground truth) to fibre orientations predicted by registering the atlas (built without the subject's data) to the subject's mDixon scan.

#### 2.4.1 FOD Prediction

To predict fibre orientations from anatomical images, the out‐of‐sample subject's mDixon scan was first registered to the population‐averaged mDixon atlas using SyN Demons with the SSD metric, mirroring the rigid, affine and non‐linear registration stages used in atlas building. The resulting displacement field was then inverted and reoriented to warp the FOD atlas and associated muscle masks into the out‐of‐sample subject's anatomical space.

#### 2.4.2 Evaluation

For the qualitative evaluation of fibre orientation prediction, we applied the same fibre tractography methods as described previously. Fibre tracts from subject‐specific DWI‐derived FODs and atlas‐predicted FODs for the out‐of‐sample subjects were visually compared.

To determine the accuracy of image registration between the subject's mDixon and the population‐averaged mDixon atlas, the Dice coefficient was calculated between the subject's mask and the atlas mask warped to subject space. Similarly, to determine the accuracy of fibre orientation prediction, the ACC and angular difference between the subject's FOD and the atlas FOD warped to subject space were calculated.

## 3 Results

We successfully constructed 11 sets of atlases of mDixon and FOD maps for the male cohort and 9 sets for the female cohort. Data derived for each atlas, including Dice coefficients, ACCs and angular differences for both the evaluation of multichannel registration performance and the fibre orientation prediction, can be found in Tables S1 and S2.

### 3.1 Multichannel Population‐Averaged Atlas and Registration Performance

**FIGURE 2:**
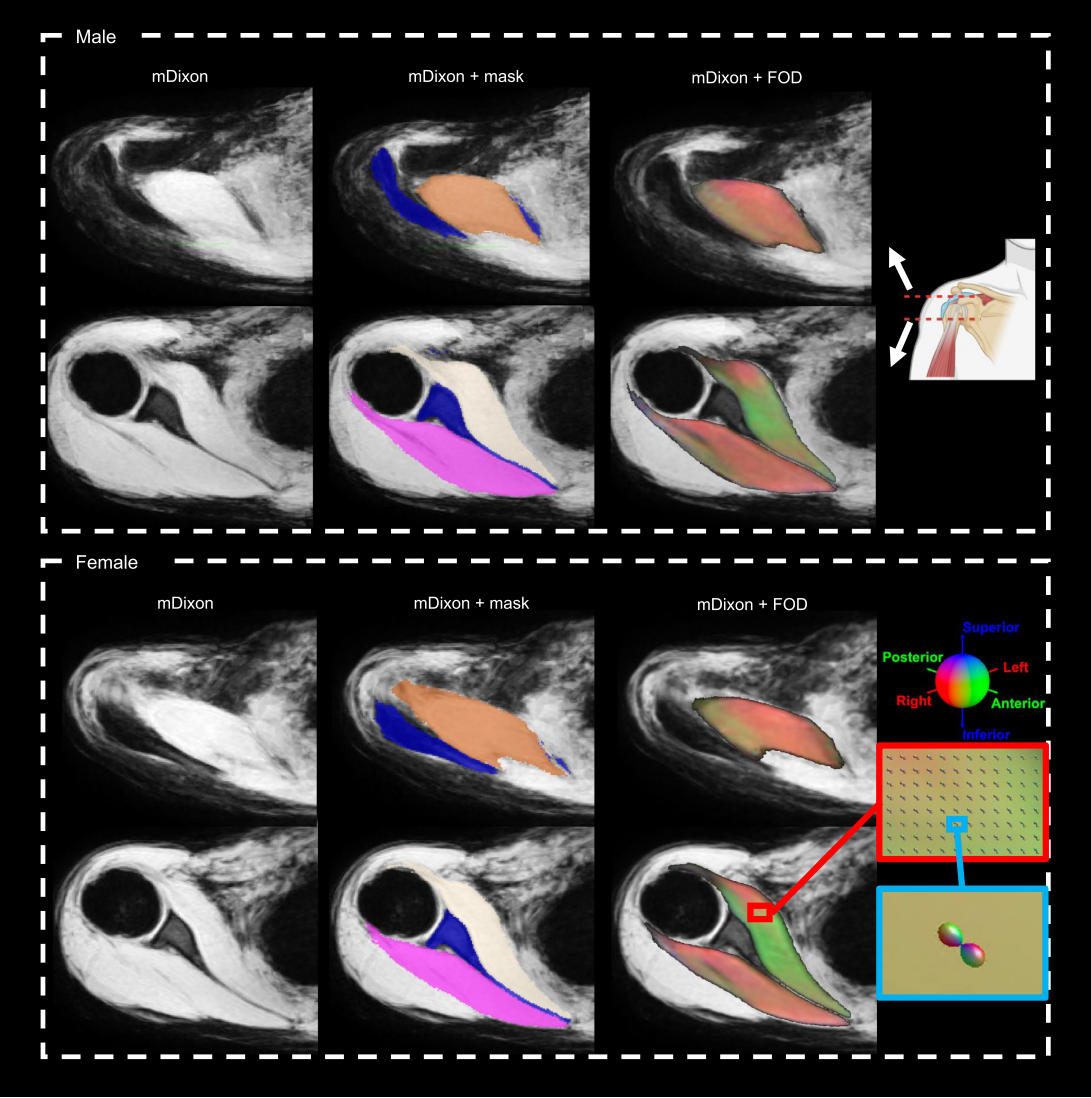
Examples of one male (top) and one female (bottom) multichannel atlas. There are two rows for the male and two rows for the female: The upper and lower rows illustrate transverse mDixon slices approximately midway through the acromioclavicular and glenohumeral joint, respectively. Shown from left to right are the mDixon water image, masks of rotator cuff muscles and scapula overlayed on mDixon image (blue, scapula; beige, subscapularis; pink, infraspinatus and teres minor; orange, supraspinatus) and an FOD image overlayed on mDixon. FODs are colour‐coded according to orientation (red: left–right; green: anterior–posterior; blue: inferior–superior).

Figure 2 presents typical examples of male and female atlases, which include mDixon, masks and FOD images. These atlases effectively preserved the anatomical information and provided good spatial alignment across the channels. The mDixon atlases, in particular, clearly defined structures such as the scapula. However, boundaries between certain muscles, specifically, the infraspinatus, teres minor and the deltoid, and the subscapularis and the serratus anterior, appeared blurry, reflecting limitations in resolving spatial details at certain intermuscular boundaries.

#### 3.1.1 Qualitative Evaluation

**FIGURE 3:**
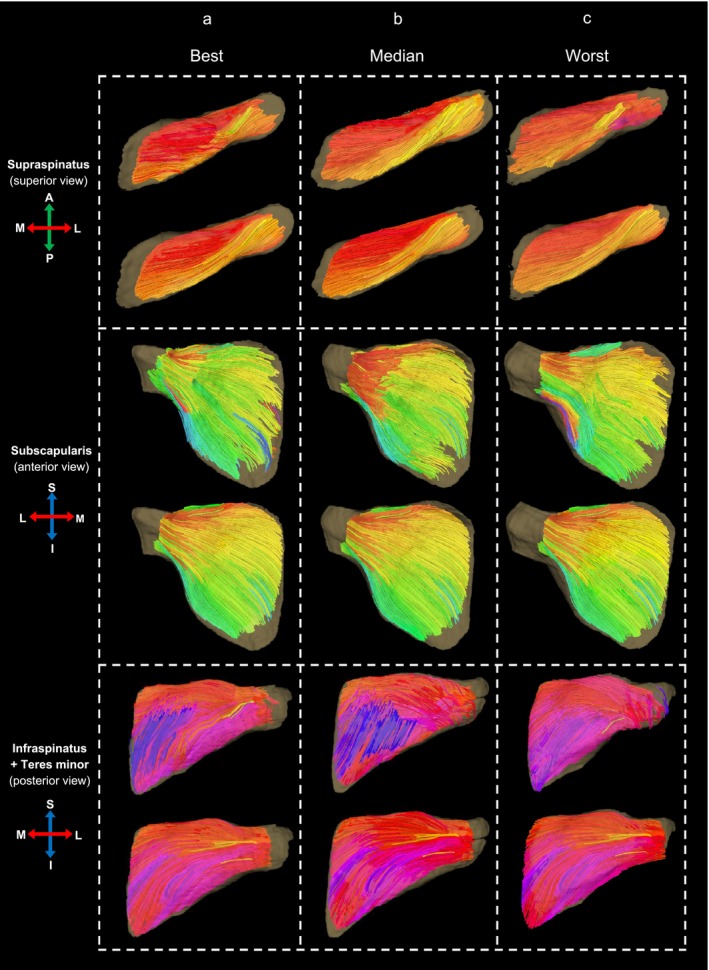
Comparison of 3D fibre tractography reconstructions of rotator cuff muscles from the right shoulder, using data from an atlas generated from 10 male subjects. For each muscle, the illustration showcases the tractographies for subjects with the (a) best, (b) median and (c) worst angular alignment within this cohort. Each pair highlights the fibre reconstructions set against the 3D surface model of the muscle in transparent yellow; the top and bottom row displays fibre reconstructed from the subject's original FODs and the FOD atlas, respectively. Fibre tracts are colour‐coded according to orientation (red: medial–lateral; green: anterior–posterior; blue: inferior–superior).

Figure 3 offers a visual representation from one atlas, showcasing the fibre tracts of subjects with the best, median and worst angular alignment, based on ACC values. The tracts displayed a transition from anatomically plausible and coherent pathways in the best cases to those characterised by noisy and implausible trajectories in the worst. Notably, fibres reconstructed from the FOD atlas demonstrated smoother tracts and extended across broader regions of the muscles. Furthermore, fibre orientations in the population‐averaged atlas closely resembled individual subject tracts.

#### 3.1.2 Quantitative Evaluation

**FIGURE 4:**
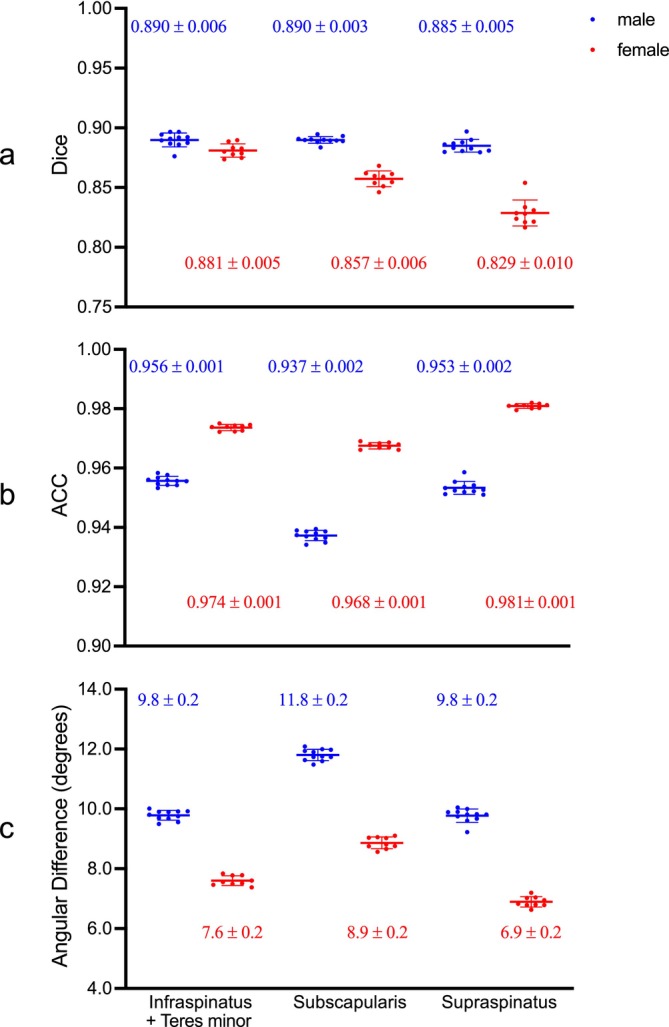
Scattergram of the registration performance metrics across different muscles for the male (*n* = 11, in blue) and female (*n* = 9, in red) cohorts. Each data point represents the average (a) Dice coefficient, (b) ACC and (c) angular difference for each muscle calculated across the *n* − 1 subjects included in constructing each atlas. The means and standard deviations for these metrics (shown as horizontal lines and error bars) are computed across atlases. Dice coefficients reflect the spatial overlap between atlas muscle segmentations and the subject's segmentations warped to the atlas space, with values closer to 1 indicating more overlap. ACCs and angular differences quantify the alignment of fibre orientation distributions, with higher ACCs and lower angular differences indicating better agreement.

There was a consistently high degree of spatial overlap and angular alignment between the images of subjects registered to the atlases and the corresponding atlases for both male and female cohorts, as evidenced by the high Dice coefficients (males: 0.888; females: 0.856; averaged across all muscles), high ACCs (males: 0.949; females: 0.974) and small angular differences (males: 10.5°; females: 7.8°; Figure 4). Small standard deviations observed across the atlases for the LOOCV (Figure 4) further indicate the high degree of consistency in registration performance. Detailed histograms illustrating the distribution of these metrics across muscles and atlases are available in Figure S1.

### 3.2 Prediction of Fibre Orientations for Out‐Of‐Sample Subjects

#### 3.2.1 Qualitative Evaluation

**FIGURE 5:**
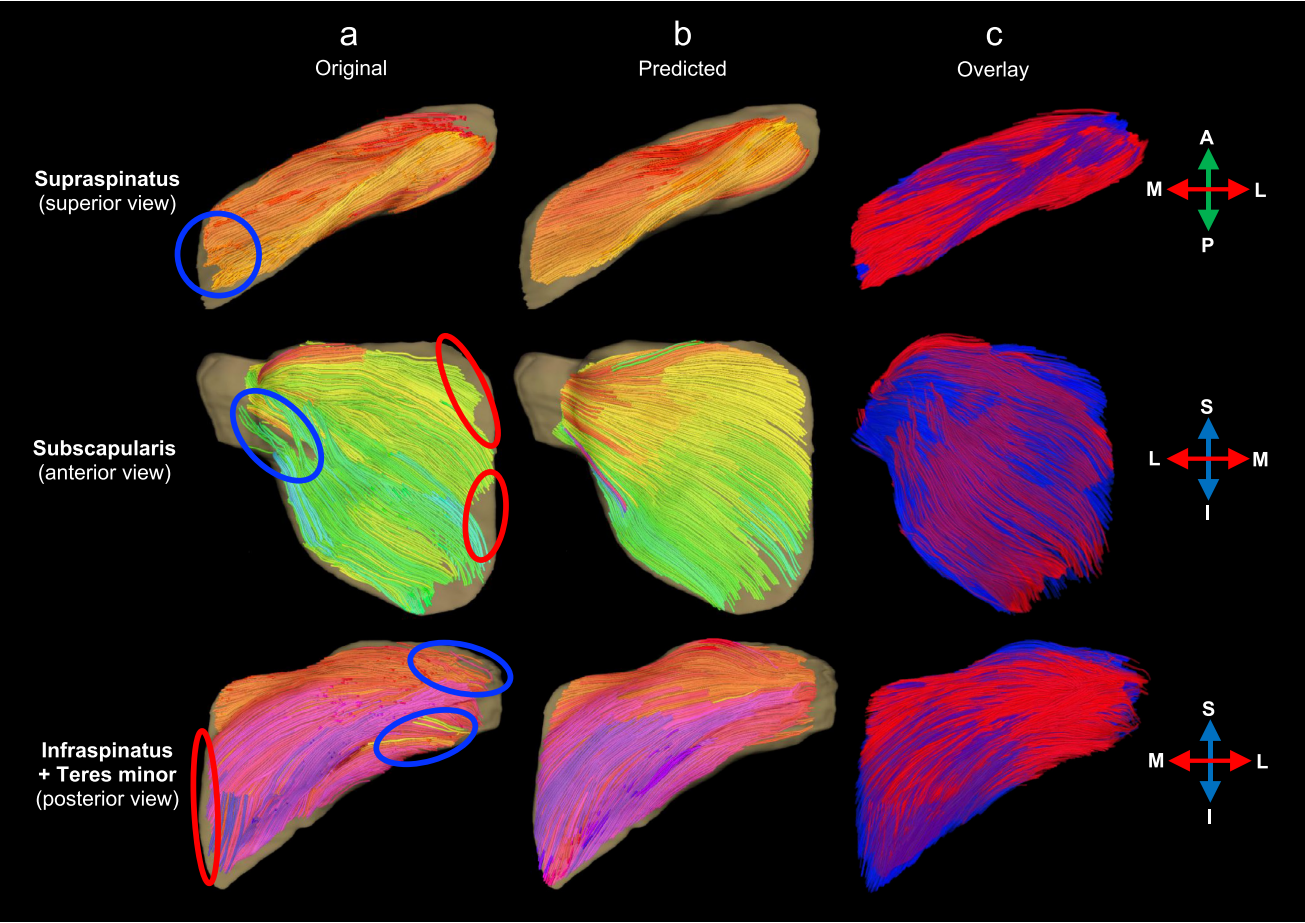
Representative 3D fibre tractography reconstructions of rotator cuff muscles from the right shoulder of an out‐of‐sample subject in the male cohort. Fibres reconstructed from the subject's (a) original and (b) predicted fibre orientations colour‐coded according to orientation (red: medial–lateral; green: anterior–posterior; blue: inferior–superior), set against 3D surface models of rotator cuff muscles in transparent yellow. In Panel (a), anatomically implausible fibre tracts and regions with missing data are highlighted with blue and red circles, respectively. Panel (c) overlays fibre from the subject's original fibre orientations (in transparent red) with those from the predicted fibre orientations (in transparent blue) for direct visual comparison.

Fibre tracts reconstructed from original and predicted fibre orientations of out‐of‐sample subjects showed a high degree of visual correspondence (Figure 5), demonstrating that the atlas can effectively predict complex patterns of 3D fibre orientations using anatomical image data alone. Fibres reconstructed from the predicted fibre orientations generally showed smoother tracts with greater coverage, particularly along the medial border of the subscapularis, infraspinatus and teres minor, compared with those from the original fibre orientations.

#### 3.2.2 Quantitative Evaluation

**FIGURE 6:**
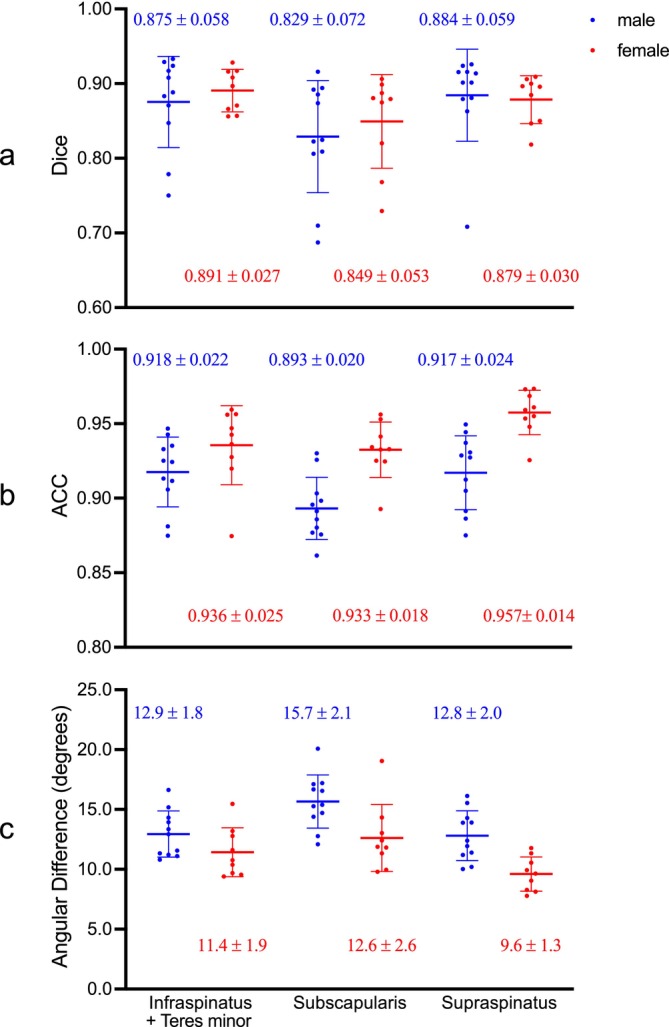
Scattergram of the evaluation metrics for out‐of‐sample prediction for the male (*n* = 11, in blue) and female (*n* = 9, in red) subjects. Each data point represents the (a) Dice coefficient, (b) ACC, and (c) angular difference calculated between the out‐of‐sample subject's FOD (ground truth) and the atlas FOD warped to subject space (prediction). The means and standard deviations for these metrics (shown as horizontal lines and error bars) are computed across subjects. Dice coefficients assess spatial alignment between atlas‐derived and subject‐specific muscle segmentations in subject space (values closer to 1 indicate better spatial alignment). ACCs and angular differences measure how well the predicted fibre orientations match those derived from the subject's DWI, with higher ACCs and lower angular differences reflecting greater accuracy in fibre orientation predictions.

**FIGURE 7:**
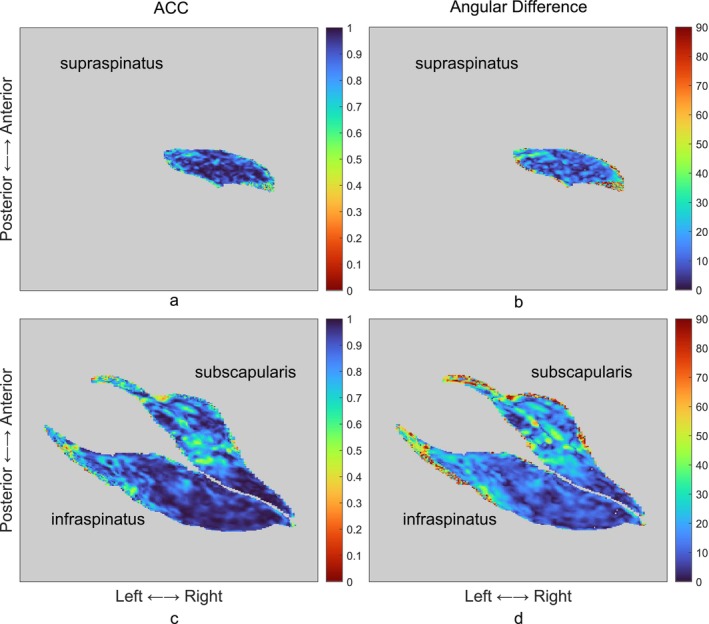
Representative visualisation of fibre orientation prediction accuracy for an out‐of‐sample subject on transverse slices. Panels (a) and (c) illustrate the ACCs for the supraspinatus (a) and the combined infraspinatus, teres minor and subscapularis (c). Panels (b) and (d) present the angular differences for the same muscle groups. The metrics were calculated between the original and predicted fibre orientations for the out‐of‐sample subject.

Both male and female atlases exhibited high Dice coefficients (males: 0.863; females: 0.873; averaged across all muscles) and ACCs (males: 0.909; females: 0.942), coupled with relatively low angular differences (males: 13.8°; females: 11.2°) (Figure 6). Variability in these metrics was noted between subjects and across muscles but remained within a relatively narrow range. Detailed histograms illustrating the distribution of these metrics across muscles and atlases are available in Figure S2. Regions of relatively low ACCs and correspondingly high angular differences, indicating regions of less satisfactory angular alignment, were most obvious at muscle boundaries and within the subscapularis (Figure 7).

## 4 Discussion

This study describes the construction of a population‐averaged atlas of human rotator cuff muscle shape and architecture, highlighting two key applications. First, we demonstrated the use of the atlas to accurately predict fibre orientations from anatomical MRI alone, bypassing the need for diffusion‐weighted scans, at least for some applications. Second, we used the atlases to smooth fibre tracts and fill in areas where fibre tracts were sparsely reconstructed because of image noise, resulting in smoother and more continuous fibre predictions derived from the atlas. However, we note that the atlas‐derived fibre orientations were not integrated back into the original DWI data to directly enhance the FOD estimation. Therefore, the observed improvement reflects smoother tractography from predicted data, rather than a direct enhancement of DWI‐based reconstructions.

Our multichannel registration pipeline was built on methods previously used in neuroimaging [24, 25, 26, 27]. One example is a study that integrated *T*_1_, *T*_2_, and FOD data from 20 neonatal brains using similar registration methods and reported a mean Dice coefficient of 0.735 and ACC of 0.455, averaged across brain tissues [25]. In comparison, our study achieved higher Dice coefficients and ACCs, likely due to less complex fibre pathways in rotator cuff muscles. Our results underscore the effectiveness of the registration pipeline in characterising interindividual differences in both the shape and internal architecture of muscles.

Despite good quantitative results, we noticed that boundaries between adjacent muscle groups in the mDixon atlases were sometimes blurry. These regions exhibited lower angular alignment, indicating relatively poor registration performance. In general, the need for multichannel registration to synthesise information about anatomical structure obtained from mDixon images with information about fibre orientation obtained from DWI presented unique challenges. For example, noise and image artifacts in DWI data could bias the registration process. Nonetheless, population‐averaged atlases had noticeably less noise and fewer artifacts than the muscle reconstructions calculated from original DWIs of individual subjects, as demonstrated by both qualitative and quantitative evaluation. Thus, population‐averaging of muscle reconstructions has a noise‐cancelling effect, as has been observed previously in brain imaging studies [47, 48]. The fidelity of our registration outputs, atlas construction, and predicted fibre orientations might be enhanced by refining the multichannel registration algorithm, for instance, by assigning greater weights to channels with higher certainty [26].

Interestingly, our results showed slightly lower Dice coefficients in the female cohort compared with males, indicating reduced spatial overlap during registration. However, this group simultaneously exhibited higher fibre orientation prediction accuracy. This apparent discrepancy may be due to differences in image quality or anatomical consistency between groups. For example, several female subjects demonstrated clearer muscle–fat contrast and more consistent intramuscular structure in their DWI scans, which may have facilitated better estimation of FODs despite imperfect spatial alignment. Further investigation is warranted to determine whether sex‐specific anatomical or imaging characteristics systematically influence prediction performance.

The multichannel registration approach and the resultant population‐averaged atlases have potentially broad applications. The atlases could serve as highly accurate mean shapes for SSMs [49], with correspondences established through the advanced registration process that integrates information from both anatomical and diffusion MRIs. This integration offers an enhanced level of detail and accuracy over traditional landmark‐based approaches and provides a more precise representation of the complex rotator cuff anatomy, which may improve predictions made with musculoskeletal models [50, 51]. Furthermore, the population‐averaged FOD atlas presents a promising approach to mitigating the challenges posed by the intrinsic sensitivity of DWI to noise and image artifacts. By leveraging the atlas's smoothed representations and expanded coverage of muscle fibres, it becomes possible to refine the analysis of subject‐specific fibre orientations. For example, the atlas could be employed as a filtering tool to selectively combine subject‐specific fibre orientations derived from DWI with the atlas information. By minimising anatomically implausible fibre tracts and enhancing tract coverage, this refined analysis may improve measurement accuracy and deepen our understanding of muscle function.

Another promising application of this approach is the prediction of fibre orientations from anatomical images when DWI data are not available. For example, predicted fibre orientations could be used in subject‐specific finite element models of muscles built from anatomical MRI scans alone, enhancing their anatomical fidelity and the accuracy of simulations [52].

### 4.1 Limitations

The generalisability of the atlases presented in this study is limited by the small number of participants and narrow demographic range of our cohort, which consisted exclusively of younger individuals without shoulder injuries. As such, the current atlases may not accurately represent muscle architecture in older populations or individuals with muscle atrophy, disease, or injury. This limits their direct applicability in some clinical contexts, such as for modelling post‐injury adaptation, age‐related degeneration or the effects of rotator cuff tears on muscle architecture. Future research should focus on expanding the demographic breadth of the atlases, incorporating data from subjects with a broader spectrum of ages, physical conditions and clinical backgrounds.

Examination of the out‐of‐sample subjects revealed that the least accurate fibre orientation predictions were associated with poorer image quality in subjects' mDixon images and partial volume effects that made identification of muscle boundaries difficult. These aspects of image quality are likely to be key factors affecting registration accuracy. Additionally, large between‐subject variations in muscle volumes, particularly evident when initially attempting to create a unified atlas for both male and female subjects, also compromised registration quality. This underscores the need for the development of more robust image registration methods to accommodate substantial anatomical variations across individuals and cohorts.

Variability in imaging protocols and equipment across different studies also poses challenges, as our registration and prediction algorithms were optimised for a specific set of MRI settings and protocols. As such, differences in equipment and scanning parameters that exist in broader clinical practice could affect the robustness and reliability of the atlases outside controlled conditions. Specifically, our current fibre orientation prediction method hinges on accurate registration of mDixon images from new subjects to these mDixon‐based atlases, where the imaging conditions were closely matched. Applying these atlases to images acquired with different protocols or modalities, such as more commonly used *T*_1_‐ or *T*_2_‐weighted MRI sequences, would require validation of the ability of our atlases to accurately align with these new modalities. Multicentre, multimodal validation studies could determine the limits and capabilities of the atlas‐based approach when applied to broader datasets.

Furthermore, although population‐averaging improves tract smoothness and reduces noise, it may also introduce bias by attenuating subject‐specific anatomical features. This trade‐off can reduce sensitivity to individual variation, which is an important consideration in applications where high anatomical fidelity is required, such as personalised modelling or surgical planning. The influence of averaging on anatomical specificity warrants further investigation and may be mitigated by hybrid approaches that selectively retain individual‐specific features.

Another limitation is that we did not compare the contribution of channels to registration performance, so similar performance may be achieved with reduced numbers of channels (e.g., using only the in‐phase mDixon image or excluding FOD channels). Adding channels improved registration performance in tongue muscles [53] and the brain [27], but future studies on rotator cuff muscles should systematically assess the contribution of channels to registration and prediction performance to identify the most efficient configurations for different use cases.

## 5 Conclusion

This study successfully demonstrated the utility of multichannel population‐averaged atlases in enhancing the reconstruction of rotator cuff muscle architecture and accurately predicting fibre orientations from anatomical MRI scans alone. These atlases, robust against noise and rich in anatomical detail, present promising tools for advancing musculoskeletal modelling and clinical diagnostics in shoulder pathologies.

## Conflicts of Interest

The authors declare no conflicts of interest.

## Supporting information


**Figure S1:** Histograms showing the distribution of metrics computed for evaluation of multichannel registration performance. The top two rows display the ACCs (upper row) and angular differences (lower row) for one example atlas within the male cohort (*n* = 10), whereas the bottom two rows present the same metrics for one example atlas from the female cohort (*n* = 8). Each line within the histograms represents the results from one subject within that atlas, with each individual subject distinguished by a unique colour.
**Figure S2:** Histograms showing the distribution of metrics computed for evaluation of fibre orientation prediction results. The top two rows display the ACCs (upper row) and angular differences (lower row) for the male cohort (*n* = 11), whereas the bottom two rows present the same metrics for the female cohort (*n* = 9). Each line within the histograms represents the results from one atlas, with each atlas distinguished by a unique colour.
**Table S1:** Dice coefficients, angular correlation coefficients (ACCs) and angular differences for evaluations of multichannel registration performance and fibre orientation prediction accuracy for the male cohort.
**Table S2:** Dice coefficients, angular correlation coefficients (ACCs) and angular differences for evaluations of multichannel registration performance and fibre orientation prediction accuracy for the female cohort. nbm70119‐sup‐0003‐Suppl_Material.docx.

## Data Availability

The data that support the findings of this study are available on request from the corresponding author. The data are not publicly available due to privacy or ethical restrictions.
