# Supplementary material for: Prediction of Rotator Cuff Muscle Fibre Orientations Using a Population‐Averaged Atlas Generated With Anatomical and Diffusion‐Weighted Magnetic Resonance Images
Source: NMR Biomed. 2025 Aug 31;38(10):e70119. doi: 10.1002/nbm.70119 (PMC12399922; doi:10.1002/nbm.70119)
Supplement: Supplementary file 1 — Figure S1: Histograms showing the distribution of metrics computed for evaluation of multichannel registration performance. The top two rows display the ACCs (upper row) and angular differences (lower row) for one example atlas within the male cohort (n = 10), whereas the bottom two rows present the same metrics for one example atlas from the female cohort (n = 8). Each line within the histograms represents the results from one subject within that atlas, with each individual subject distinguished by a unique colour. Figure S2: Histograms showing the distribution of metrics computed for evaluation of fibre orientation prediction results. The top two rows display the ACCs (upper row) and angular differences (lower row) for the male cohort (n = 11), whereas the bottom two rows present the same metrics for the female cohort (n = 9). Each line within the histograms represents the results from one atlas, with each atlas distinguished by a unique colour. Table S1: Dice coefficients, angular correlation coefficients (ACCs) and angular differences for evaluations of multichannel registration performance and fibre orientation prediction accuracy for the male cohort. Table S2: Dice coefficients, angular correlation coefficients (ACCs) and angular differences for evaluations of multichannel registration performance and fibre orientation prediction accuracy for the female cohort. nbm70119‐sup‐0003‐Suppl_Material.docx. [file NBM-38-e70119-s001.docx]

**Table S1.** Dice coefficients, angular correlation coefficients (ACCs), and angular differences for evaluations of multi-channel registration performance and fibre orientation prediction accuracy for the male cohort.

| Evaluation task | Atlas number | Infraspinatus + Teres minor | | | Subscapularis | | | Supraspinatus | | |
| --- | --- | --- | --- | --- | --- | --- | --- | --- | --- | --- |
|  |  | Dice | ACC | Angular difference (º) | Dice | ACC | Angular difference (º) | DC Dice | ACC | Angular difference (º) |
| Multi-channel registration performance ^a^ | 1 | 0.886  (0.036) | 0.955  (0.010) | 9.9  (1.3) | 0.890  (0.034) | 0.938  (0.014) | 11.7  (1.4) | 0.897  (0.026) | 0.954  (0.018) | 9.8  (1.8) |
|  | 2 | 0.892  (0.033) | 0.957  (0.010) | 9.6  (1.1) | 0.889  (0.035) | 0.935  (0.012) | 12.0  (1.4) | 0.886  (0.026) | 0.952  (0.017) | 9.8  (1.8) |
|  | 3 | 0.887  (0.032) | 0.954  (0.010) | 9.9  (1.3) | 0.889  (0.037) | 0.939  (0.013) | 11.6  (1.4) | 0.888  (0.028) | 0.953  (0.020) | 9.8  (1.9) |
|  | 4 | 0.876  (0.034) | 0.953  (0.009) | 10.0  (1.2) | 0.895  (0.027) | 0.939  (0.013) | 11.5  (1.1) | 0.880  (0.032) | 0.952  (0.018) | 9.9  (1.8) |
|  | 5 | 0.892  (0.033) | 0.956  (0.010) | 9.9  (1.1) | 0.884  (0.033) | 0.939  (0.013) | 11.7  (1.4) | 0.881  (0.030) | 0.955  (0.016) | 9.7  (1.7) |
|  | 6 | 0.891  (0.030) | 0.958  (0.010) | 9.5  (1.1) | 0.890  (0.034) | 0.939  (0.013) | 11.6  (1.5) | 0.887  (0.031) | 0.959  (0.012) | 9.2  (1.0) |
|  | 7 | 0.897  (0.034) | 0.956  (0.010) | 9.7  (1.2) | 0.891  (0.040) | 0.937  (0.012) | 11.8  (1.5) | 0.883  (0.031) | 0.951  (0.019) | 10.0  (1.8) |
|  | 8 | 0.887  (0.037) | 0.956  (0.010) | 9.8  (1.2) | 0.889  (0.037) | 0.937  (0.014) | 11.9  (1.5) | 0.890  (0.023) | 0.952  (0.017) | 9.9  (1.8) |
|  | 9 | 0.897  (0.030) | 0.956  (0.009) | 9.8  (1.1) | 0.893  (0.038) | 0.934  (0.013) | 12.1  (1.6) | 0.880  (0.027) | 0.951  (0.017) | 10.0  (1.7) |
|  | 10 | 0.894  (0.033) | 0.958  (0.008) | 9.7  (1.1) | 0.888  (0.038) | 0.937  (0.015) | 12.0  (1.5) | 0.883  (0.027) | 0.953  (0.019) | 9.8  (1.9) |
|  | 11 | 0.890  (0.028) | 0.954  (0.008) | 9.9  (1.1) | 0.890  (0.030) | 0.936  (0.014) | 11.9  (1.5) | 0.881  (0.033) | 0.954  (0.018) | 9.6  (1.7) |
| Fibre orientation prediction ^b^ | 1 | 0.888 | 0.935 | 11.2 | 0.874 | 0.891 | 15.3 | 0.879 | 0.891 | 16.1 |
|  | 2 | 0.871 | 0.913 | 13.4 | 0.710 | 0.903 | 14.4 | 0.901 | 0.929 | 11.2 |
|  | 3 | 0.924 | 0.943 | 11.1 | 0.892 | 0.886 | 17.1 | 0.926 | 0.944 | 10.2 |
|  | 4 | 0.933 | 0.947 | 10.8 | 0.823 | 0.862 | 20.1 | 0.915 | 0.937 | 11.4 |
|  | 5 | 0.929 | 0.925 | 11.3 | 0.916 | 0.877 | 16.7 | 0.914 | 0.905 | 13.9 |
|  | 6 | 0.917 | 0.906 | 14.3 | 0.894 | 0.876 | 16.6 | 0.924 | 0.886 | 15.5 |
|  | 7 | 0.847 | 0.912 | 13.9 | 0.806 | 0.926 | 12.8 | 0.881 | 0.950 | 10.0 |
|  | 8 | 0.779 | 0.881 | 15.2 | 0.687 | 0.880 | 17.2 | 0.708 | 0.875 | 14.3 |
|  | 9 | 0.883 | 0.924 | 12.9 | 0.885 | 0.930 | 12.1 | 0.916 | 0.931 | 12.4 |
|  | 10 | 0.750 | 0.875 | 16.6 | 0.825 | 0.896 | 15.4 | 0.863 | 0.927 | 11.9 |
|  | 11 | 0.908 | 0.933 | 11.6 | 0.809 | 0.898 | 14.7 | 0.901 | 0.912 | 13.9 |

^a^ Values are means (standard deviations) of the median values of these metrics calculated for every subject included in each atlas (n = 10).

^b^ Values are the median values of these metrics for the out-of-sample subject of each atlas (n = 1).

**Table S2.** Dice coefficients, angular correlation coefficients (ACCs), and angular differences for evaluations of multi-channel registration performance and fibre orientation prediction accuracy for the female cohort.

| Evaluation task | Atlas number | Infraspinatus + Teres minor | | | | Subscapularis | | | | Supraspinatus | | | |
| --- | --- | --- | --- | --- | --- | --- | --- | --- | --- | --- | --- | --- | --- |
|  |  | Dice | ACC | Angular difference (º) | Dice | | ACC | Angular difference (º) | Dice | | ACC | Angular difference (º) |  |
| Multi-channel registration performance ^a^ | 1 | 0.879  (0.040) | 0.973  (0.006) | 7.8  (1.0) | 0.859  (0.045) | | 0.966  (0.007) | 9.0  (1.4) | 0.821  (0.073) | | 0.980  (0.005) | 7.2  (1.0) |  |
|  | 2 | 0.883  (0.037) | 0.974  (0.006) | 7.5  (1.0) | 0.855  (0.042) | | 0.969  (0.007) | 8.7  (1.2) | 0.817  (1.249) | | 0.981  (0.004) | 6.8  (0.9) |  |
|  | 3 | 0.878  (0.033) | 0.974  (0.006) | 7.6  (1.1) | 0.860  (0.041) | | 0.967  (0.006) | 9.1  (1.2) | 0.821  (0.074) | | 0.981  (0.005) | 6.9  (1.0) |  |
|  | 4 | 0.875  (0.045) | 0.972  (0.006) | 7.8  (1.0) | 0.846  (0.047) | | 0.968  (0.007) | 8.8  (1.4) | 0.831  (0.067) | | 0.982  (0.005) | 6.8  (1.1) |  |
|  | 5 | 0.883  (0.035) | 0.974  (0.006) | 7.5  (1.0) | 0.854  (0.038) | | 0.969  (0.007) | 8.6  (1.2) | 0.824  (0.067) | | 0.982  (0.005) | 6.6  (1.0) |  |
|  | 6 | 0.880  (0.040) | 0.974  (0.006) | 7.6  (1.0) | 0.861  (0.044) | | 0.968  (0.007) | 8.8  (1.3) | 0.829  (0.074) | | 0.981  (0.006) | 6.8  (1.3) |  |
|  | 7 | 0.874  (0.029) | 0.972  (0.005) | 7.8  (0.7) | 0.851  (0.033) | | 0.966  (0.006) | 9.1  (1.1) | 0.828  (0.072) | | 0.981  (0.005) | 6.8  (1.0) |  |
|  | 8 | 0.890  (0.032) | 0.975  (0.006) | 7.4  (0.9) | 0.868  (0.038) | | 0.967  (0.007) | 9.0  (1.3) | 0.854  (0.037) | | 0.980  (0.005) | 7.0  (1.1) |  |
|  | 9 | 0.889  (0.033) | 0.975  (0.006) | 7.5  (1.0) | 0.862  (0.043) | | 0.968  (0.008) | 8.8  (1.4) | 0.834  (0.063) | | 0.980  (0.006) | 7.0  (1.2) |  |
| Fibre orientation prediction ^b^ | 1 | 0.856 | 0.943 | 10.8 | 0.768 | | 0.941 | 11.3 | 0.847 | | 0.953 | 8.3 |  |
|  | 2 | 0.866 | 0.956 | 9.5 | 0.887 | | 0.934 | 13.0 | 0.906 | | 0.969 | 9.1 |  |
|  | 3 | 0.857 | 0.936 | 12.8 | 0.729 | | 0.953 | 9.8 | 0.818 | | 0.973 | 7.8 |  |
|  | 4 | 0.897 | 0.947 | 10.4 | 0.879 | | 0.933 | 12.4 | 0.896 | | 0.955 | 10.5 |  |
|  | 5 | 0.871 | 0.920 | 13.2 | 0.820 | | 0.933 | 14.3 | 0.850 | | 0.961 | 11.8 |  |
|  | 6 | 0.917 | 0.956 | 9.7 | 0.906 | | 0.925 | 11.8 | 0.897 | | 0.959 | 9.9 |  |
|  | 7 | 0.916 | 0.959 | 9.4 | 0.899 | | 0.956 | 10.0 | 0.909 | | 0.973 | 8.1 |  |
|  | 8 | 0.928 | 0.928 | 11.6 | 0.875 | | 0.925 | 11.9 | 0.885 | | 0.925 | 9.6 |  |
|  | 9 | 0.908 | 0.875 | 15.5 | 0.881 | | 0.893 | 19.1 | 0.900 | | 0.948 | 11.3 |  |

^a^ Values are means (standard deviations) of the median values of these metrics calculated for every subject included in each atlas (n = 8).

^b^ Values are the median values of these metrics for the out-of-sample subject of each atlas (n = 1).

**Figure S1.** Histograms showing the distribution of metrics computed for evaluation of multi-channel registration performance. The top two rows display the ACCs (upper row) and angular differences (lower row) for one example atlas within the male cohort (n = 10), while the bottom two rows present the same metrics for one example atlas from the female cohort (n = 8). Each line within the histograms represents the results from one subject within that atlas, with each individual subject distinguished by a unique colour.

**Figure S2.** Histograms showing the distribution of metrics computed for evaluation of fibre orientation prediction results. The top two rows display the ACCs (upper row) and angular differences (lower row) for the male cohort (n = 11), while the bottom two rows present the same metrics for the female cohort (n = 9). Each line within the histograms represents the results from one atlas, with each atlas distinguished by a unique colour.
